# A hospital based survey to evaluate knowledge, awareness and perceived barriers regarding breast cancer screening among females in Bangladesh

**DOI:** 10.1016/j.heliyon.2020.e03753

**Published:** 2020-04-10

**Authors:** Mohammad Nurul Amin, Md. Giash Uddin, Md. Nazim Uddin, Md. Zahedur Rahaman, Shafayet Ahmed Siddiqui, Md. Shahadat Hossain, Md. Rakibul Islam, Md. Nazmul Hasan, S.M. Naim Uddin

**Affiliations:** a Department of Pharmacy, Atish Dipankar University of Science and Technology, Dhaka 1230, Bangladesh; b Pratyasha Health Biomedical Research Center, Dhaka 1230, Bangladesh; c Department of Pharmacy, Noakhali Science and Technology University, Noakhali 3814, Bangladesh; d Department of Pharmacy, University of Chittagong, Chittagong 4331, Bangladesh

**Keywords:** Cancer research, Epidemiology, Health sciences, Public health, Clinical research, Education, Breast cancer, Awareness, Knowledge, Barriers, Females, Bangladesh

## Abstract

**Background:**

Early diagnosis of breast cancer is essential for mitigating its related morbidity and mortality. Therefore, high awareness is required.

**Objectives:**

We aimed to evaluate the knowledge, awareness and perceived barriers among females in Bangladesh regarding breast cancer.

**Methods:**

A hospital-based survey was performed from April 2019 to June 2019. A total of 500 females aged >18 years were recruited to the study. The participants were selected by trained personnel and physicians via simple random sampling.

**Results:**

The mean participant age was 37.13 ± 12.66 years. Among all the participants, 79% were married, 4% were single, 3% were divorced and 14% were widowed. We observed that 80.6% of respondents were housewives, 5% were students and 14.4% were working women. The participants had a severe lack of knowledge and awareness, and perceived barriers regarding breast cancer screening. Breast cancer was more linked to personal history, occupation and, marital status. Shyness, fear, lack of knowledge and deficient awareness programs were the major perceived barriers.

**Conclusion:**

Educational interventions and proper, appropriate and socially acceptable awareness programs will help to ameliorate knowledge and awareness by addressing barriers regarding breast cancer among the females in Bangladesh.

## 1 Introduction

Cancer is an abnormal development of cells that initially remains localized; with time, it metastasizes, leading to malignant tumour [1]. It is the second-leading cause of death worldwide; it was responsible for 9.6 million deaths reported in 2018 and accounts for about one in six deaths worldwide. Around 70% of deaths from cancer occur in low- and middle-income countries [2]. Among all cancers, breast cancer is the most common in women [3, 4]. In 2018, the World Health Organization (WHO) reported that approximately 627,000 women died from breast cancer, representing 15% of total cancer death among women [5, 6].

Countries in South Asia are facing a secret epidemic of breast cancer. There is a worrying rise in the incidence of breast cancer. Around 588 million women aged >15 years are facing an increasing epidemic of breast cancer. Throughout South Asia, knowledge of epidemiology, genetic and various environmental contexts of breast cancer is scarce. There are no central cancer registries that can deliver complete nationwide data [7]. In Pakistan, one in nine women develop breast cancer, and the breast cancer mortality rate is 26.76% [1, 8]. In India, breast cancer incidence was 27% and the mortality rate was 21.5% [9].

Bangladesh is a small country with the highest population density in the world. The increasing population and scarcity of proper knowledge may lead to an increased number of patients with cancer, which is inevitable. Breast cancer is also increasing at an alarming rate. A recent report suggested that the breast cancer incidence rate in Bangladesh was 22.5 per 100,000 women [10]. The mean age of patients with breast cancer is 41.8 years, among which reproductive-age women account for >56%. This represents a higher proportion of premenopausal cases in Bangladeshi patients with breast cancer. It might be due to missing cases involving older women who feel nervous about pursuing medical aid and who have lower treatment preferences than younger women in Bangladesh [7]. Moreover, along with the high incidence rate, around 90% of breast cancers are diagnosed at stage III–IV [11]. This rate of late-stage diagnosis is alarming and is becoming quite frequent. This could be due to the severe lack of knowledge, no health insurance policy and lack of awareness of breast self-screening methods for detecting breast cancer at home, with the increased prevalence of breast cancer [8]. Another issue is the cultural norms. Sometimes, it is difficult to use words such as ‘breasts’ in public. Furthermore, the cancer registry in Bangladesh is not functioning well. There is an urgent need to understand the cancer burden in this country [12]. According to the Breast Health Global Initiative (BHGI), if females are well-equipped with knowledge and awareness of breast cancer self-examination (BSE), the disease can be diagnosed at an early stage, and disease management can be easier [13].

However, Bangladesh has paid little attention to the knowledge and awareness of breast cancer in females. There is a lack of trained oncologists to serve the huge number of patients with breast cancer. Besides, there are only a few studies on breast cancer in the Bangladeshi population, but the disease burden has remained immense, as cases are identified late. Moreover, information from previous studies does not reflect the complete scenario of Bangladeshi communities and has many limitations. Thus, there is a compelling need for a more comprehensive study on this issue. Therefore, considering the socio-cultural norms and other facts, we designed a hospital-based survey to explore the complete scenario of knowledge, awareness and perceived barriers of breast cancer in Bangladesh among females.

## 2 Materials and methods

### 2.1 Study design and sampling

The National Institute of Cancer Research & Hospital (NICRH), a tertiary care hospital, was selected for this hospital-based survey. The hospital is in Dhaka, the capital city of Bangladesh, where people of all ethnicities come to seek medical treatment for cancer [3]. All the participants of this study were females who had been randomly selected between April 2019 to June 2019. The sample size was calculated following the method of Masood et al. [14]. Considering allowable error and non-response rate, some extra samples were taken over the calculated sample size for proper analysis and maximum validity. Finally, a total of 500 participants were selected for this study. We used simple random sampling, and the lottery method was used to select the participants [3]. Then, each participant was assigned a unique identification number and was selected randomly. The inclusion criteria were: willingness to participate in the study, age >18 years, ability to understand English or Bengali, and patients with breast cancer. Participants who did not match the above criteria were excluded from the study. A written consent form was collected from the participants. The study protocol was approved by the NICRH ethical research committee (reference no: DO-NICRH/2018/44).

### 2.2 Study questionnaire

The questionnaire was constructed combining previously published articles [14, 15, 16, 17, 18]. The questionnaire comprised four segments: A. Demographic characteristics of respondents; B. General knowledge of participants regarding breast cancer; C. Awareness of breast cancer symptoms and risk factors; D. Barriers towards breast cancer screening. The questionnaire was first written in English and then translated into Bengali for a better understanding by the participants. The questionnaire was validated by a panel of experts that included oncologists, clinical pharmacists, social science graduates, university professors with expertise in relevant fields and healthcare professionals. A few participants were first included to determine if there were any difficulties in understanding the questionnaire; no difficulties were reported. The medical terminologies were explained to the participants in face-to-face interviews.

### 2.3 Data collection

Data collection was assisted by four dedicated volunteers who were pharmacy graduates and physicians. They had completed a 1-month training course on breast cancer screening and risk assessment and were trained on the research questionnaire. They had also attended the seminars on breast cancer awareness and perspectives in Bangladesh arranged by different medical institutions. The data collection comprised four steps. In the first step, the participants were required to fill out the questionnaire, including questions related to socio-demographic and anthropometric information. The questionnaire was distributed to the participants, to whom we explained the research purpose and that their confidentiality would be maintained. The authors and trained volunteers were present to clarify any doubts or questions the participants might have had. The questionnaires were completed and collected immediately thereafter.

### 2.4 Statistical analysis

All collected data were entered into Microsoft Excel 2013 and transferred to SPSS software version 22.0 for Windows. The data were analysed using descriptive statistics to summarize the demographic data and responses of the participants.

## 3 Results

**Figure 1:**
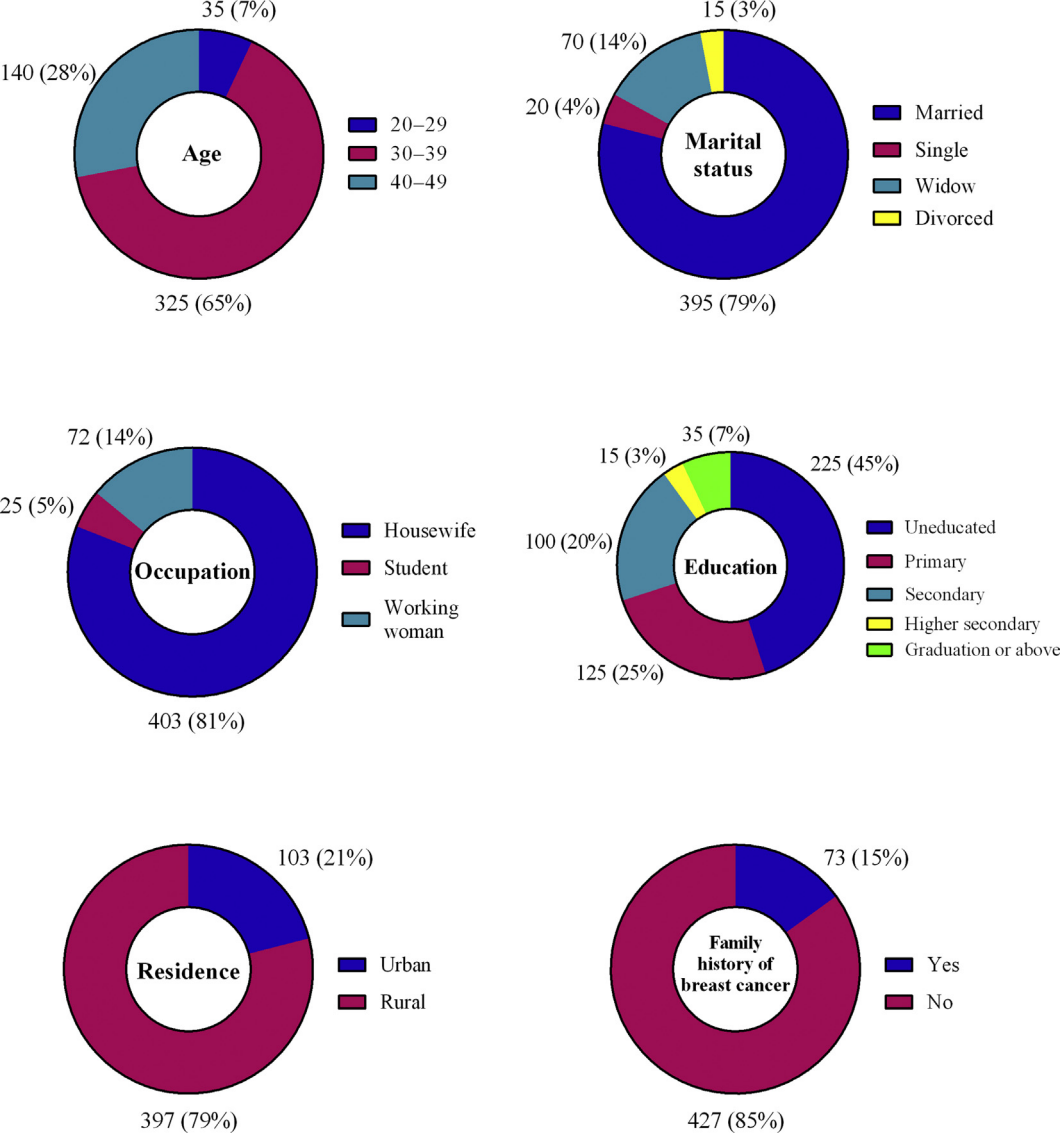
Demographic characteristics of respondents (n = 500).

Overall, 500 female patients with breast cancer participated in the study, yielding a response rate of 94.55%. Figure 1 shows the participants’ demographic characteristics. The mean participant age was 37.13 ± 12.66 years. The majority (79%) of participants were married, 4% were single, 3% were divorced and 14% were widowed. Most of the participants (81%) were housewives. The majority (45%) were uneducated and only 25% had primary-level education. Most of the participants were from rural areas (79%). Of these, none had a prior personal history of breast cancer, and 85.40% had no breast cancer family history.

When evaluating general knowledge regarding breast cancer, 64.4% of participants reported that they had some knowledge of breast cancer. Of the total participants, 17.2% reported that they did not know about the BSE and how it was done. About 91% of participants had never heard of any breast cancer screening programs, and 9% of participants considered breast cancer an infrequent disease. Table 1 shows the participants’ responses to their general knowledge regarding breast cancer.

**Table 1:** General Knowledge of respondents regarding breast cancer.

Statements	Yes	No
Have you ever heard about breast cancer?	327 (64.4%)	173 (34.6%)
Is breast cancer a rare disease?	45 (9.0%)	455 (91.0%)
Do you know how to perform breast self-examination?	86 (17.2%)	414 (82.8%)
Have you heard about screening programs?	45 (9.0%)	455 (91.0%)

Table 2 summarises the participants’ awareness of breast cancer symptoms and risk factors. Of breast cancer, 27.6% of the participants reported a change in nipple position, 57.8% had pain in one breast or armpit, 11% had nipple dimpling, 62% had breast redness, 71.4% had a lump under the armpit and 39% had altered breast size and shape. The participants were also queried about the risk factors of breast cancer. Here, all participants had individual genetic make-up and increased age. Of the participants, 66% had avoided breastfeeding, 17.4% had a painless breast lump, 5.2% had borne their first child at age >30 years, 65.4% had started menarche aged <11 years and 90% frequently used oral contraceptive pills.

**Table 2:** Awareness in females about the symptoms and risk factors of breast cancer.

Statements	Yes	No	Don't know
Symptoms of breast cancer			
1. Changed nipple position	138 (27.6%)	362 (72.4%)	
2. Pulling in of nipple		500 (100.0%)	
3. Pain in one of breasts or armpit	289 (57.8%)	211 (42.2%)	
4. Dimpling of breast skin	55 (11.0%)	445 (89.0%)	
5. Discharge or bleeding from nipple	26 (5.2%)	474 (94.8%)	
6. Lump or thickening in breast		500 (100.0%)	
7. Redness of breast skin	310 (62.0%)	190 (38.0%)	
8. Lump or thickening under armpit	357 (71.4%)	143 (28.6%)	
9. Changes in the size and shape	195 (39.0%)	305 (61.0%)	
**Risk factors of breast cancer**			
1. Genetic makeup of individual	500 (100.0%)		
2. Advancing age	500 (100.0%)		
3. Avoiding breast feeding	330 (66.0%)	165 (33.0%)	5 (1.0%)
4. Painless lump or thickening in breast	87 (17.4%)	413 (82.6%)	
5. First childbirth at age above 30 years	26 (5.2%)	474 (94.8%)	
6. Null parity	5 (1.0%)	460 (92.0%)	35 (7.0%)
7. Menarche below 11 years	327 (65.4%)	173 (34.6%)	
8. Frequent use of oral contraceptive pills	450 (90.0%)	20 (4.0%)	30 (6.0%)
9. Trauma/breast injury	49 (9.8%)	451 (90.2%)	
10. Late menopause	412 (82.4%)	48 (9.6%)	40 (8.0%)
11. Hormone replacement therapy		500 (100.0%)	
12. High fats intake or obesity	21 (4.2%)	479 (95.8%)	

Finally, it was important to determine the possible barriers to breast screening. Many participants felt embarrassed about telling others about breast cancer (64.6%) and had no idea what other people, i.e. those in society, thought about it (62.2%). Of these respondents, 53% were concerned about stigma following their breast cancer diagnosis, 53.6% felt shy about uncovering their breasts, 41.8% were afraid of hospitals and health facilities, 56.6% worried about what the doctor might find during screening and 52.2% had difficulty talking with the doctor. Most of the participants had a lack of knowledge (58.6%) and were afraid of undergoing a mammography (60.8%), an important technique for diagnosing breast cancer. Moreover, 38.6% of the participants were busy or had no time for screening, and 53.4% reported deficient awareness programs (Table 3).

**Table 3:** Barriers towards breast cancer screening.

Barriers	Yes	No	Don't know
1. Acceptable to touch my body	217 (43.4%)	283 (56.6%)	
2. Embarrassing to tell people about	323 (64.6%)	177 (35.4%)	
3. No idea about what other people think	311 (62.2%)	189 (37.8%)	
4. Stigma following the diagnosis of cancer	265 (53.0%)	235 (47.0%)	
5. Feeling shy to uncover my breasts	268 (53.6%)	232 (46.4%)	
6. Fear of hospitals and health facilities	209 (41.8%)	291 (58.2%)	
7. Feeling worried about what a doctor might find	283 (56.6%)	191 (38.2%)	26 (5.2%)
8. Difficulty talking to doctor	261 (52.2%)	218 (43.6%)	21 (4.2%)
9. Lack of knowledge	293 (58.6%)	155 (31.0%)	52 (10.4%)
10. Fear of physicians and examiners	171 (34.2%)	329 (65.8%)	
11. Afraid of having mammography	304 (60.8%)	149 (29.8%)	47 (9.4%)
12. Busy, no time to do it	193 (38.6%)	307 (61.4%)	
13. Awareness program are deficient	267 (53.4%)	163 (32.6%)	70 (14.0%)

## 4 Discussion

This hospital-based survey was carried out to determine the knowledge, awareness and perceived barriers of breast cancer among females in Bangladesh. We identified key factors that may shape comprehensive studies on breast cancer awareness. Despite the participants having some level of awareness regarding breast cancer, most of the women lacked diagnostic intervention for socio-cultural reasons. We found that the mean age of patients with breast cancer in the Bangladeshi population is comparatively lower than that of India, Pakistan and the western countries. This early age has great implications regarding the lack of knowledge and awareness of breast cancer among females in this country. Our findings are consistent with that of studies in Iran and Pakistan [14, 19, 20]. Our findings reveal the limited knowledge and lack of preventive measures for creating breast cancer awareness. We also found that knowledge of how BSE is performed is comparatively higher in India, Pakistan, and Turkey, although very much lower percentages of women were able to perform BSE [14, 21]. This clarifies why breast cancer is frequently reported at the later stages in Bangladesh. The situation is again better in Saudi Arabia, the United Arab Emirates and Ethiopia, where most women know how to perform BSE [15, 22, 23].

Most of the participants tended to avoid breastfeeding, used contraceptive pills and had late menopause, which is considered risk factors of breast cancer. In addition, breast cancer awareness was comparatively higher with age and increased level of education. This could be due to their level of education and experience-sharing with other females such as friends, relatives, and colleagues. Moreover, single female had comparatively lower breast cancer awareness than the other participants. This could be due to their younger age or less interest in sharing personal experiences with other females, as it is not a common social norm in Bangladesh. Pakistan has a similar scenario [15].

As breast cancer can be diagnosed early by breast screening programs, it was important to determine the possible barriers to screening. The results indicate that embarrassment was the leading barrier to breast screening programs. From the results, it was also evident that most of the participants were worried, lacked knowledge and were less confident about sharing their problems with healthcare providers and people in society, which is consistent with some previous studies [17, 18]. This finding may also reflect the situation in society, where there is a gap between the affected females and other people. If others in society received proper counselling, awareness programs, education and knowledge regarding breast cancer, it would mitigate the issue of late diagnosis and facilitate the proper management of patients diagnosed with breast cancer.

The findings from the present study show that a lack of knowledge of performing BSE and socio-cultural factors against breast screening might be the causes of the unsatisfactory level of breast cancer awareness in Bangladesh. More awareness programs and proper knowledge delivery may change this scenario, although breast cancer prevalence is on the rise. Healthcare professionals and the government can come forward together to formulate an executable policy that can diagnose and increase awareness among the females as well as in society. According to the American Cancer Society (ACS), BSE is helpful in early diagnosis but can increase stress and anxiety in females and decrease survival [24]. Again, in developing countries, there is a lack of interest in undergoing mammography because of the cost. Moreover, if mammography is available, mammography-based screening is not executed well [25]. Therefore, it is important to have programs on knowledge and awareness of breast cancer and BSE by targeting high-risk populations. This would help to reduce the frequency of disease and enable early-stage diagnosis and its management using the country's available resources.

One of the main limitations of the present study is that it is not representative of the whole Bangladeshi population or the participants are comparatively more health-aware. As the socio-cultural situation is not conducive to covering a wide variety of demographic classes, the study was conducted in a cancer hospital located in the nation's capital. Also, the research was conducted with no external funding. So, we were limited to collecting the sample from other regions of Bangladesh. Further research can be performed on a wider scale, covering more respondents from different demographic classes and geographic locations to picture the total scenario that may lead to the design of intervention programs on breast cancer awareness.

## 5 Conclusion

The majority of female respondents were generally unaware, lacked knowledge and awareness, and perceived barriers regarding breast cancer. Breast cancer was more linked to personal history, occupation, and marital status. Shyness, fear, lack of knowledge and awareness program deficiency were the major perceived barriers. Our findings suggest that proper, appropriate and socially acceptable awareness programs will help to improve knowledge and awareness, and address barriers regarding breast cancer among females in Bangladesh.

## Declarations

### Author contribution statement

M.N. Amin: Conceived and designed the experiments; Analyzed and interpreted the data.

M.G. Uddin: Conceived and designed the experiments; Analyzed and interpreted the data; Wrote the paper.

M.N. Uddin, M.Z. Rahaman and M.S. Hossain: Performed the experiments; Contributed reagents, materials, analysis tools or data.

S.A. Siddiqui, M.N. Hasan, S.M.N. Uddin: Analyzed and interpreted the data; Contributed reagents, materials, analysis tools or data.

M.R. Islam: Analyzed and interpreted the data.

### Funding statement

This research did not receive any specific grant from funding agencies in the public, commercial, or not-for-profit sectors.

### Competing interest statement

The authors declare no conflict of interest.

### Additional information

No additional information is available for this paper.
